# Pharmacokinetics of gallic acid and protocatechuic acid in humans after dosing with Relinqing (RLQ) and the potential for RLQ-perpetrated drug–drug interactions on organic anion transporter (OAT) 1/3

**DOI:** 10.1080/13880209.2021.1934039

**Published:** 2021-06-18

**Authors:** Ziqiang Li, Xi Du, Yanfen Li, Ruihua Wang, Changxiao Liu, Yanguang Cao, Weidang Wu, Jinxia Sun, Baohe Wang, Yuhong Huang

**Affiliations:** a Second Affiliated Hospital of Tianjin University of Traditional Chinese Medicine, Tianjin, PR China; b Tianjin Institute of Pharmaceutical Research, Tianjin, PR China; c UNC Eshelman School of Pharmacy, University of North Carolina, Chapel Hill, NC, USA; d TIPR Pharmaceutical Responsible Co., Ltd, Tianjin, PR China

**Keywords:** Herb-drug interactions (HDI), pharmacokinetic markers (PK-markers), organic anion transporter, Chinese patent medicine

## Abstract

**Context:**

Relinqing granules (RLQ) are being used alone or in combination with antibacterial drugs to treat urological disorders.

**Objective:**

This study investigates the pharmacokinetics of RLQ in humans and the potential for RLQ-perpetrated interactions on transporters.

**Materials and methods:**

Twelve healthy subjects (six women and six men) participated to compare single- and multiple-dose pharmacokinetics of RLQ. In the single-dose study, all 12 subjects received 8 g of RLQ orally. After a 7-d washout period, the subjects received 8 g of RLQ for seven consecutive days (t.i.d.) and then a single dose. Gallic acid (GA) and protocatechuic acid (PCA) in plasma and urine samples were analysed using LC-MS/MS. The transfected cells were used to study the inhibitory effect of GA (50–5000 μg/L) and PCA (10–1000 μg/L) on transporters OAT1, OAT3, OCT2, OATP1B1, P-gp and BCRP.

**Results:**

GA and PCA were absorbed into the blood within 1 h after administration and rapidly eliminated with a half-life of less than 2 h. The mean peak concentrations of GA (102 and 176 μg/L) and PCA (4.54 and 7.58 μg/L) were lower in males than females, respectively. The 24 h urine recovery rates of GA and PCA were about 10% and 5%, respectively. The steady-state was reached in 7 d without accumulation. GA was a potent inhibitor of OAT1 (IC_50_ = 3.73 μM) and OAT3 (IC_50_ = 29.41 μM), but not OCT2, OATP1B1, P-gp or BCRP.

**Discussion and conclusions:**

GA and PCA are recommended as PK-markers in RLQ-related pharmacokinetic and drug interaction studies. We should pay more attention to the potential for RLQ-perpetrated interactions on transporters.

## Introduction

Urinary tract infections (UTIs) are among the most frequent bacterial infections acquired in hospitals and communities (Geerlings 2016; Millner and Becknell 2019). UTIs represent at least 40% of all hospital-acquired infections (Tandogdu and Wagenlehner 2016). While treatment of UTIs with antibiotics is very likely to clear bacteriuria leading to a rapid resolution of clinical symptoms, the current high level of bacterial resistance development threatens this treatment of UTIs. The increasing resistance to broad-spectrum antibiotics is a particular challenge due to their overconsumption (Tamadonfar et al. 2019). Therefore, there is an urgent need to develop strategies to combat resistance through the careful use of available antibiotics or alternative therapies (Klein and Hultgren 2020; Qindeel et al. 2021).

**Figure 1.**
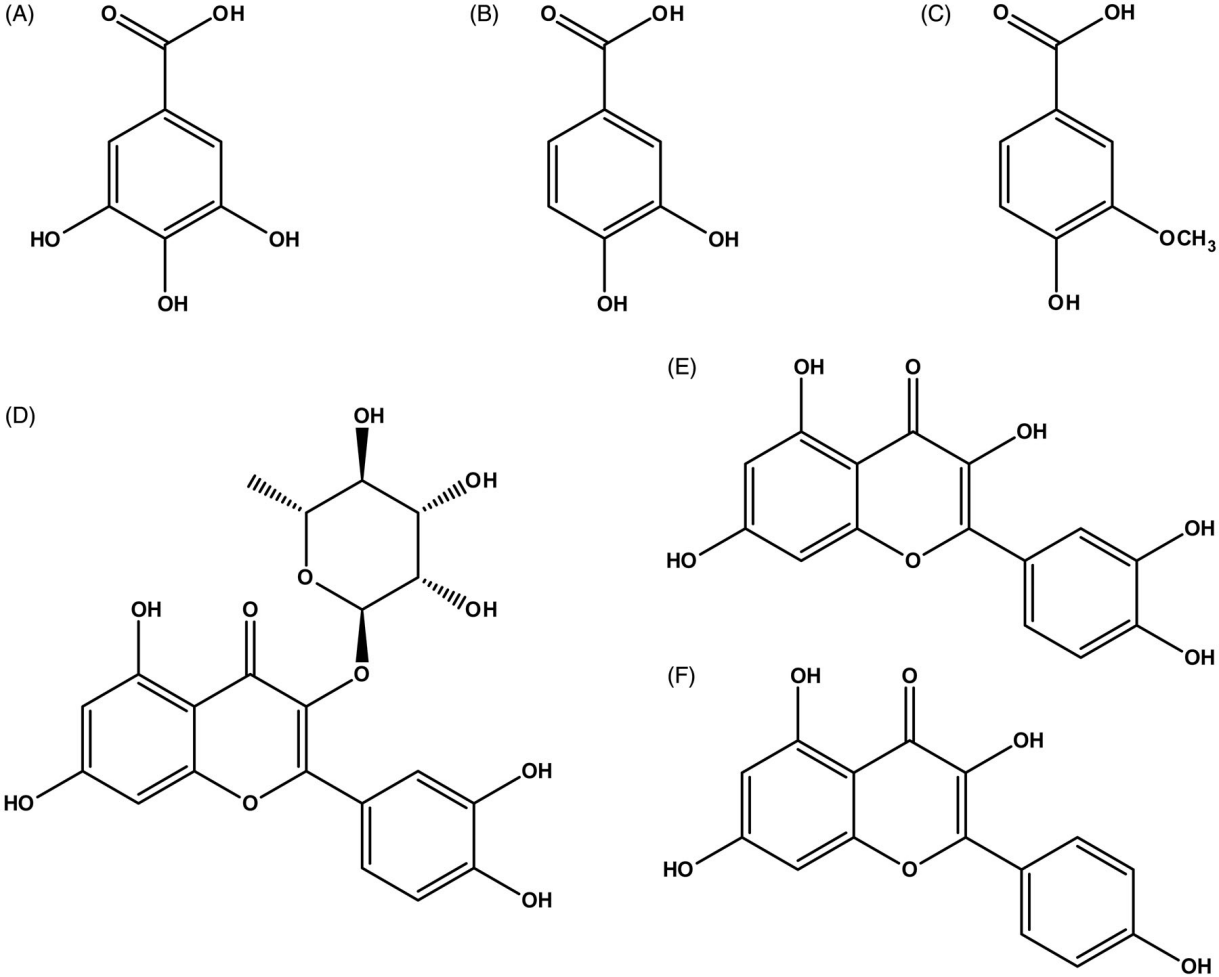
Chemical structures of (A) gallic acid, (B) protocatechuic acid, (C) vanillic acid, (D) quercitrin, (E) quercetin and (F) kaempferol.

Numerous botanical medicines have been proposed as alternative therapies for urological infections in many countries (Benevides Bahiense et al. 2017; Kim et al. 2019). Relinqing granules (RLQ), a Chinese herbal medicine, are widely used to treat various urologic disorders, including UTIs, pyelonephritis and urinary calculus (Chinese Pharmacopoeia Commission 2020). The phenolic acids and flavonoids (Figure 1) are believed to be responsible for most of their pharmacological effects (Liao et al. 2011; Zhang et al. 2017). Knowledge of the pharmacokinetic properties of RLQ is necessary to understand its clinical efficacy and toxicity. Gallic acid (GA) and protocatechuic acid (PCA) are the top two constituents of the phenolic acids contained in RLQ (Liao et al. 2013; Huang et al. 2019), and their anti-inflammatory and antibacterial activities are consistent with the main pharmacological effects of RLQ. Circulating GA and PCA have shown a relatively high exposure after oral administration of RLQ in rats (Ma et al. 2016; Huang et al. 2019). Despite the prevalence of UTIs and RLQ treatment, little is known about the pharmacokinetics of its active ingredients in humans.

The combination of RLQ and conventional therapies has created a need to understand better the risks and benefits, especially the interaction of herbs and drugs (Ren et al. 2019; Zhang et al. 2019; Yang et al. 2020). Evidence-based herbal drug interaction research is expected to become a necessary evaluation for the rational use of RLQ with other drugs prescribed for the same indications (Lu et al. 2016). The CYP450 enzyme- and transporter-mediated drug interactions are being increasingly reported in new drug development and clinical medication (USFDA 2020). RLQ displayed a significant inductive effect on the expression of CYP2C9 and CYP3A4 when used as a cocktail (Zheng et al. 2014). In contrast to metabolizing enzymes, there are different membrane transporter levels in different body tissues, which play essential roles in drug absorption, distribution and elimination. Hence, it is necessary to conduct additional evidence-based research into the transporter-mediated interactions between RLQ and concomitant drugs.

This study attempts to address the above issues by investigating the pharmacokinetics and transporter-based inhibition kinetics of the phenolic compounds. The PK-markers of RLQ in plasma and urine were identified following single and multiple RLQ doses to healthy humans. The potential effect of drug transporters on safety makes it essential to determine whether GA and PCA can affect other drugs’ absorption and disposition. The index substrates’ future results will provide essential information on the potential herb-drug interactions when using combined therapy.

## Materials and methods

### Chemicals

RLQ was kindly provided by Guizhou Warmen Pharmaceutical Co., LTD (Guiyang, China). GA, PCA, quercitrin, quercetin, kaempferol and chloramphenicol were purchased from the National Institute for Food and Drug Control (Beijing, China). Rifampicin, probenecid, verapamil, cimetidine, rhodamine 123, dimethyl sulfoxide (DMSO) and lucifer yellow were obtained from Sigma-Aldrich (St. Louis, MO). ^14^C-*para*-Amino hippuric acid (^14^C-PAH), ^3^H-estrone sulphate ammonium salt (^3^H-ES) and ^c^(^14^C-TEA) were purchased from TRC Inc. (Toronto, Canada). Dulbecco’s modified Eagle’s medium (DMEM), Hanks’ balanced salt solution (HBSS), 4-(2-hydroxyethyl) piperazine-1-ethane sulphonic acid (HEPES), Dulbecco’s phosphate-buffered saline (DPBS) and foetal bovine serum (FBS) were obtained from Gibco Life Technologies (Grand Island, NY). Acetonitrile, methanol and formic acid were purchased from Thermo Fisher Scientific (Waltham, MA). Other reagents were commercially available and of analytical grade.

### Chemical analysis

RLQ (0.2 g) was dissolved in 25 mL of methanol-water (1:1, v/v). After sonication for 1 h, the mixture was centrifuged for 10 min at 13,000 *g* and filtrated through a 0.22 µm membrane before analysis. The characteristic fingerprint of RLQ was detected using a high-performance liquid chromatography-ultraviolet (HPLC-UV) method. The contents of GA, PCA, quercitrin, quercetin and kaempferol were measured with a validated liquid chromatography-tandem mass spectrometry (LC-MS/MS) method (Tang et al. 2013). Chromatographic separations were performed using an ACQUITY UPLC BEH C18 column (2.1 mm × 100 mm, 1.7 μm) maintained at 30 °C. A gradient elution programme (0.3 mL/min) was used with the mobile phase consisting of 0.1% formic acid aqueous solution (A) and 0.1% formic acid acetonitrile (B) as follows: 5%–35% B (0–1.5 min), 95%–5% B (3–4 min) and 5% B (5 min). The injection volume was 2 µL. Mass spectrometric detections were performed on an AB Sciex Triple Quad 5500 MS equipped with an ESI interface. The analytes were monitored in MRM mode with transitions of *m/z* 169.0→125.1, 153.1→109.0, 449.1→303.1, 303.2→153.0 and 287.1→95.0 for GA, PCA, quercitrin, quercetin and kaempferol, respectively.

### Subjects

Healthy male and female volunteers aged 18–55 years with a body mass index of 19–24 kg/m^2^ were eligible for recruitment. Additional inclusion criteria included good general health as confirmed by a medical history review, a physical examination, clinical laboratory tests and a non-smoking status. Subjects were excluded if they had any allergies, haematological abnormalities or a history of renal or hepatic diseases. Subjects were also excluded if they had recently taken any medications or ingested grapefruit juice, St. John’s wort or agents that interact with RLQ for at least 2 weeks before dosing and during the study. All subjects provided written informed consent before enrolment.

### Clinical trial design

The study protocol was approved by the Ethics Committee of the Second Affiliated Hospital of Tianjin University of Traditional Chinese Medicine. This study was conducted to assess single- and multiple-dose pharmacokinetics of RLQ in healthy male and female subjects (ChiCTR-OPh-16010029). Subjects were offered standard meals at 4 and 10 h after dosing. Water was not permitted during the hour before and the hour after dosing, except for 240 mL administered with dosing; additional water intake was allowed at all other times.

In the single-dose study, 8 g of RLQ was administered orally on day 1. A 7-d washout time was designed between single-dose and multiple-dose pharmacokinetic studies. In the multiple-dose study, subjects received multiple doses of RLQ (8 g, t.i.d.) for seven consecutive days from day 8 to day 14 and a single dose of RLQ (8 g) on the morning of day 15. On days 1 and 15, blood samples (3 mL) were collected from the antecubital vein catheter before drug administration and at 0.08, 0.17, 0.33, 0.5, 0.75, 1, 1.5, 2, 3, 4, 6, 8, 12 and 24 h after dosing. Additional pre-dose samples were collected in the morning on days 12, 13 and 14. Urine samples were collected over a 12 h interval before dosing and at the following time intervals thereafter: 0–2, 2–5, 5–8, 8–12 and 12–24 h. All samples were stored at −80 °C until analysis.

### Safety and tolerability

Safety was monitored by performing clinical laboratory tests, 12-lead ECGs and physical examinations at baseline and scheduled times. All adverse events recorded during the study were coded according to the Medical Dictionary for Regulatory Activities version 15.1 (McLean, VA).

### Sample preparation

All analytes were extracted from plasma and urine samples by simple protein precipitation extraction method using acetonitrile. Briefly, 5 µL of internal standard solution and 300 μL of acetonitrile were added to the spiked blank matrix (100 μL) and vortexed for 3 min and then centrifuged at 13,000 *×* *g* for 10 min. The supernatant was transferred and dried using a flow of nitrogen. The residue was re-dissolved in 100 µL of 10% methanol solution containing 0.1% formic acid. An aliquot of 5 µL solution was injected into the LC-MS/MS system for analysis.

### GA and PCA assay

The GA and PCA concentrations were determined using a validated LC-MS/MS method with some modifications (Ma et al. 2015). The chromatographic separation was performed on an Acquity Ultra Performance LC instrument (Waters, Milford, MA) with a gradient programme in an ACQUITY UPLC BEH C_18_ column (2.1 mm × 100 mm, 1.7 μm). The gradient programme was performed with the mobile phase (0.3 mL/min) consisting of 0.1% formic acid aqueous solution (A) and acetonitrile (B) as follows: 97% A (0–8.5 min), 60% A (8.7–11 min), 10% A (11.5–13.5 min) and 97% A (14–15 min). The injection volume was 2 µL. The mass spectrometry analysis was performed on an AB Sciex Triple Quad 5500 MS (Framingham, MA) equipped with an ESI source. The quantitative analyses of GA, PCA and chloramphenicol were performed in a negative mode by multiple reaction monitoring (MRM) with the ion transitions of *m/z* 169.0→125.1, 153.1→109.0 and 321.1→152.1, respectively.

The analytical method’s validation was conducted according to the FDA’s Bio-analytical Method Validation guidance (USFDA 2018). Blank samples were obtained from six subjects for selectivity. The calibration curves were assessed by plotting the peak response ratio against the nominal concentrations of GA (1.00, 2.00, 10.0, 40.0, 100, 200, 320 and 400 μg/L) and PCA (0.50, 1.00, 5.00, 20.0, 50.0, 100, 160 and 200 μg/L). The low, medium and high QC concentrations were 3.00, 60.0 and 300 μg/L and 1.50, 30.0 and 150 μg/L for GA and PCA, respectively. Intra- and inter-day accuracy and precision were measured using six replicates of QCs in three separate batches. QCs’ acceptance criteria were ± 15% SD of nominal values, and the precision was ± 15% RSD, except for LLOQ ± 20% SD. The matrix effect and recovery were investigated using six lots of blank matrix from individual sources. Stability was assessed by spiking low and high QCs in blank samples using four conditions: for post-preparative stability (24 h at 4 °C), bench-top stability (24 h at 25 °C), long-term stability (3 months at −80 °C) and freeze-thaw stability (for three cycles).

### Pharmacokinetic analysis

Pharmacokinetic parameters of GA and PCA were estimated with a non-compartmental method using Phoenix WinNonlin version 6.4 (Certara, Princeton, NJ). The peak plasma concentration (*C*_max_) and time to reach the peak plasma concentration (*T*_max_) were calculated from the actual plasma concentration data. The area under the plasma concentration-time curve from zero to the last measurable concentration (*AUC*_0–_*_t_*) was calculated *via* the linear trapezoidal rule. *AUC*_0–∞_ was calculated using the following formula: *AUC*_0–∞_ = *AUC*_0–_*_t_* + *C*_t_/*k*_e_, where *C_t_* is the last plasma concentration measured. The terminal elimination half-life (*t*_1/2_) was calculated as 0.693/*k*_e_, and the total clearance (*CL/F*) was calculated as dose/*AUC*_0–∞_. The accumulation index was calculated as *AUC*_ss_/*AUC*_0–_*_t_*.

### Cell culture

The human multidrug resistance protein (MDR)1-transfected Madin–Darby Canine Kidney (MDCK) cells, human breast cancer resistance protein (BCRP)-transfected MDCK cells, human organic anion transporter (OAT)1-transfected MDCK cells, OAT3-transfected second portion of proximal tubule (S2) cells, human organic cation transporter (OCT)2-transfected S2 cells, human organic anion transporting polypeptide (OATP)1B1-transfected human embryonic kidney 293 (HEK293) cells and their MOCK cells were kindly provided by Japan Fuji Biomedical Co., Ltd. (Osaka, Japan). Cells were cultured in DMEM supplemented with 10% FBS, 100 units/mL penicillin and 100 units/mL streptomycin. All cells were incubated in a humidified atmosphere of 95% air and 5% CO_2_ at 37 °C. A solution of 0.25% trypsin-EDTA was used to detach the cells from flasks. Cell culture flasks, 24-well cell culture plates, and transwell polycarbonate inserts (12 mm diameter, 0.4 μm pore size) were purchased from Corning Co-star Corp. (Bedford, MA).

### Inhibition of cellular uptake in S2-OAT3, S2-OCT2, HEK293-OATP1B1 and MDCK-OAT1 cells

Concentration-dependent inhibitions were conducted to study GA and PCA’s inhibitory effects on uptake transporters OAT1, OAT3, OCT2 and OATP1B1. The MDCK-OAT1, S2-OAT3, S2-OCT2 and HEK293-OATP1B1 cell lines were utilized in this study. The uptake experiments were adapted from previously reported methods with a few modifications (Akimitsu et al. 2010; Chiba et al. 2013; Wang et al. 2013; Li et al. 2014). Briefly, S2-OAT3, S2-OCT2 and S2-mock cells were trypsinized and uniformly suspended in the culture medium’s designated volume to provide a density of 2.0 × 10^5^ cells/mL. A 1.0-mL aliquot of the cell suspension was added into each well of 24-well insert plates. HEK293-OATP1B1, MDCK-OAT1 and their mock cells were seeded in 24-well plates at a density of 1.5 × 10^5^ cells/well. After incubating for 48 h, the cells were washed twice with preheated DPBS and then pre-incubated with DPBS for 10 min at 37 °C. An aliquot of 500 µL DPBS containing radio-labelled probe substrates was added to initiate the uptake in the presence or absence of GA (50, 150, 500, 1500 and 5000 μg/L), PCA (10, 30, 100, 300 and 1000 μg/L) or positive inhibitors at 37 °C for the designated uptake time. The probe substrates and positive inhibitors were ^14^C-PAH (5 µM) and probenecid (100 µM), ^3^H-ES (0.05 µM) and cimetidine (600 µM), ^14^C-TEA (5 µM) and probenecid (100 µM), ^3^H-ES (0.05 µM) and rifampicin (60 µM) for OAT1, OAT3, OCT2 and OATP1B1, respectively. Three washes terminated the experiment with ice-cold DPBS buffer after removing the incubation buffer. Cells were lysed with 400 μL of 0.1 mM sodium hydroxide solution. The cell lysate and 3.0 mL of aquasol-2 scintillation fluid were put into a scintillation flask. The radioactive intensity was measured using a Tri-Carb 2910TR scintillator (PerkinElmer, Waltham, MA). The final concentration of DMSO in the assay was less than 0.1%. All experiments were performed in triplicate.

### Inhibition of cellular efflux in MDCK-MDR1 and MDCK-BCRP cells

Inhibitory effects of GA and PCA on efflux transporters MDR1 and BCRP were investigated with the fluorogenic substrates rhodamine 123 (10 µM) and lucifer yellow (10 µM), respectively. The positive control inhibitors of MDR1 and BCRP were verapamil (10 µM) and cyclosporine (20 µM), respectively. The efflux experiments were adapted from previously reported methods with minor modifications (Reznicek et al. 2017). Briefly, MDCK-MDR1, MDCK-BCRP and MDCK-mock cells were trypsinized and uniformly suspended in the designated volume of HBSS to provide a density of 4.0 × 10^5^ cells/mL. For 0.5 mL of cell suspension and 1.5 mL of HBSS buffer medium were added in the apical and basolateral compartments of 12-well Transwell plates. After cultivation for 7 d, cells were washed twice and equilibrated for 30 min at 37 °C with the pre-warmed HBSS buffer. The integrity of cell monolayers was verified by the determination of the value of transepithelial electrical resistance (TEER) using a Millipore Millicell-ERS system (Millipore Corporation, Bedford, MA) (200–300 Ω × cm^2^). An aliquot of 1.5 mL transport buffer containing probe substrates was added in the basolateral compartment to initiate the efflux in the presence or absence of GA (50, 150, 500, 1500 and 5000 μg/L), PCA (10, 30, 100, 300 and 1000 μg/L) or positive inhibitors at 37 °C. An aliquot of 0.5 mL empty transport buffer was placed in the apical compartment. After 2 h of co-incubation, 100 μL of HBSS in the apical compartment was transferred to 96-well plates. Fluorescence was measured at 485 nm (excitation) and 546 nm (emission) for rhodamine 123, and 425 nm (excitation) and 528 nm (emission) for Lucifer yellow. The final concentration of DMSO in the assay was less than 0.1%. All experiments were performed in triplicate.

### Statistical analysis

All data were presented as means ± SD. Statistical analyses were performed using SPSS software version 22 (Chicago, IL). Differences between groups were compared using Student’s *t*-test. *p* < 0.05 was considered statistically significant.

## Results

### Chemical analysis

**Figure 2.**
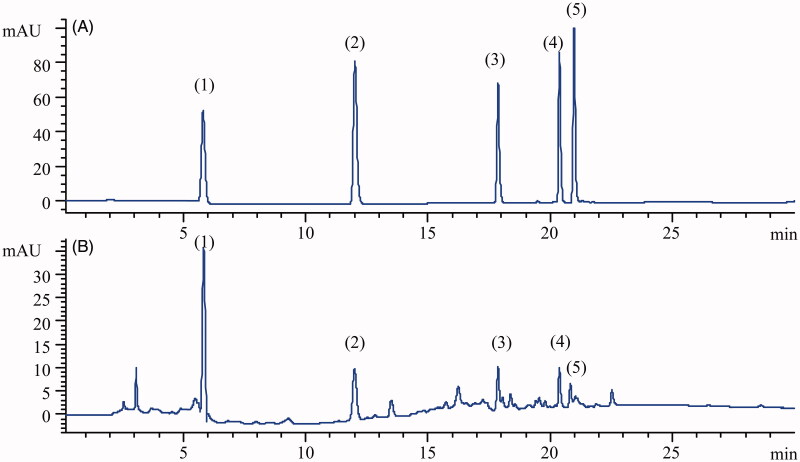
Typical chromatograms of (1) gallic acid, (2) protocatechuic acid, (3) quercitrin, (4) quercetin and (5) kaempferol obtained from an analysis of standard solution (A) and RLQ solution (B).

The HPLC-UV fingerprint of RLQ was detected and established at the 254 nm wavelength. Five characteristic peaks were identified by comparison with the references (Figure 2). The relative retention times of GA, PCA, quercitrin, quercetin and kaempferol were 5.86, 11.95, 17.82, 20.32 and 21.04 min, respectively. According to the label, the specification of RLQ is 4 g. The quantitative determination of the five phenolic markers was implemented by using an LC-MS/MS instrument. The GA, PCA, quercitrin, quercetin and kaempferol contents were 25.32, 1.25, 1.62, 0.21 and 0.13 mg, respectively. It showed that the GA content in this batch meets the Chinese Pharmacopoeia requirements, stating that the level of GA in RLQ should not be less than 23.0 mg (Chinese Pharmacopoeia Commission 2020).

### Assay validation

**Figure 3.**
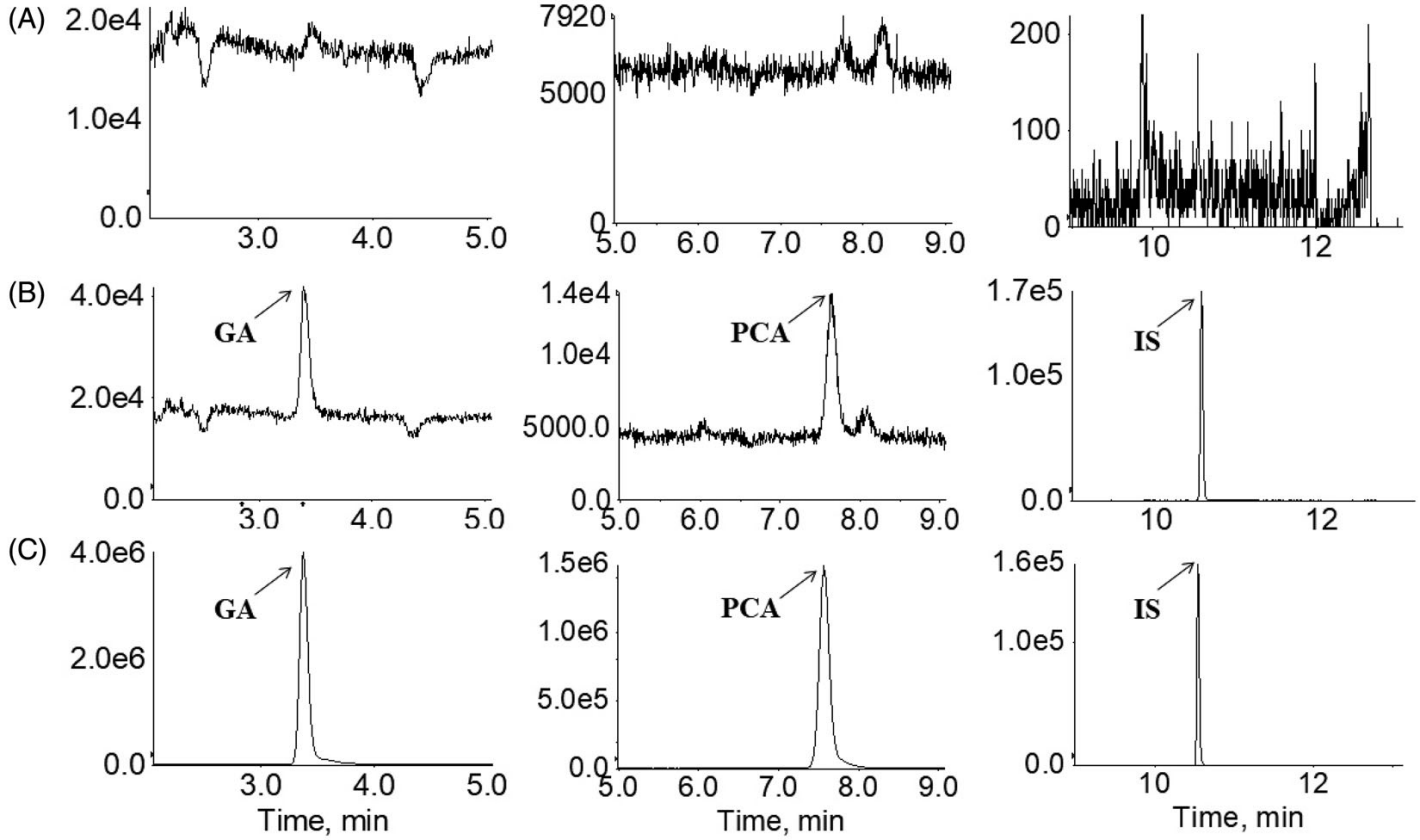
Multiple reaction monitoring chromatograms of (**A**) blank plasma; (B) blank plasma spiked with a standard mixture of gallic acid (GA, 1) and protocatechuic acid (PCA, 2) at the LLOQ level with internal standard (IS, 3); (C) human plasma sample taken 2 h following the oral administration of RLQ to human subjects.

The typical MRM chromatogram showed no peak interfering at the retention times of GA, PCA and IS in blank plasma samples, proving a reasonable specificity (Figure 3). GA and PCA’s calibration curves exhibited good linearity over the concentration range of 1.00–400.0 and 0.50–200.0 μg/L, respectively. The lower limit of quantification (LLOQ) of GA and PCA were 1.00 and 0.50 ng/mL, respectively. The intra-day precision and accuracy of quality control samples ranged from 0.95% to 9.06% and −7.41% to −2.98%, respectively. The inter-day precision and accuracy of quality control samples were in the range of 1.74–9.98% and −8.54% to −3.14%, indicating that this method is within the acceptable range for precision and accuracy (Table 1). The extraction recovery and matrix effect were in the range of 91.08–99.79% and 101.39–111.65%, respectively. The stability of GA and PCA in the biological matrix were validated under various storage conditions, including room temperature for 24 h, frozen at −80 °C for 3 months, three freeze-thaw cycles and the post-preparative stability at 4 °C for 24 h (Table 2).

**Table 1.** Accuracy, precision, recovery and matrix effect for analysis of gallic acid (GA) and protocatechuic acid (PCA) in quality control samples (*n* = 6).

Analyte	Spiked concentration (μg/L)	Intra-day		Inter-day		Recovery (%, means ± SD)	Matrix effect (%, means ± SD)
		Accuracy (%)	Precision (%)	Accuracy (%)	Precision (%)		
GA	1.00	−5.13	7.04	−3.14	9.36	–	–
	3.00	−3.62	1.53	−3.98	2.62	99.22 ± 3.62	109.27 ± 5.77
	60.0	−2.98	2.44	−3.38	3.13	92.49 ± 2.01	103.11 ± 0.51
	300.0	−7.41	0.95	−6.87	1.93	91.08 ± 2.37	102.36 ± 0.47
PCA	0.50	−4.29	9.06	−8.54	9.98	–	–
	1.50	−6.93	2.46	−7.01	4.01	99.79 ± 3.34	111.65 ± 8.18
	30.0	−5.56	2.07	−5.03	2.63	92.49 ± 2.45	101.39 ± 0.51
	150.0	−5.54	1.42	−6.39	1.74	92.26 ± 2.21	101.40 ± 0.74

**Table 2.** Stability of gallic acid (GA) and protocatechuic acid (PCA) in quality control samples (*n* = 3).

Analyte	Spiked concentration (μg/L)	Post-preparative		Bench-top		Long-term		Three freeze-thaw cycles	
		Stability (%)	RSD (%)	Stability (%)	RSD (%)	Stability (%)	RSD (%)	Stability (%)	RSD (%)
GA	3.00	88.48	2.15	98.11	6.99	99.70	2.30	85.89	2.76
	300.0	94.37	5.00	94.81	8.21	93.68	2.33	93.17	3.83
PCA	1.50	90.60	3.85	97.69	2.96	99.04	2.15	87.22	4.42
	150.0	96.93	5.21	95.44	7.48	93.67	2.35	94.22	3.79

### Demographic characteristics

Twelve healthy subjects (six males and six females) were enrolled in the pharmacokinetic study of RLQ. Their age, height, weight, and body mass index were 27.0 ± 1.94 (24.0–30.0) years, 1.65 ± 0.08 (1.57–1.81) m, 58.6 ± 6.27 (50.2–70.1) kg and 21.5 ± 1.37 (19.5–23.3) kg/m^2^, respectively (Table 3). All 12 recruited subjects were healthy native Chinese individuals whom all completed the study protocol as planned. No major protocol deviations were identified, and no serious side effects were observed throughout the study.

**Table 3.** Demographic characteristics of healthy subjects in this study (*n* = 12).

Characteristic	Mean ± SD (range)
Male/female	6/6
Age (years)	27.0 ± 1.94 (24–30)
Height (m)	1.65 ± 0.08 (1.57–1.81)
Weight (kg)	58.6 ± 6.27 (50.2–70.1)
BMI (kg/m^2^)	21.5 ± 1.37 (19.5–23.3)

### Plasma GA and PCA profiles of RLQ in human subjects

**Figure 4.**
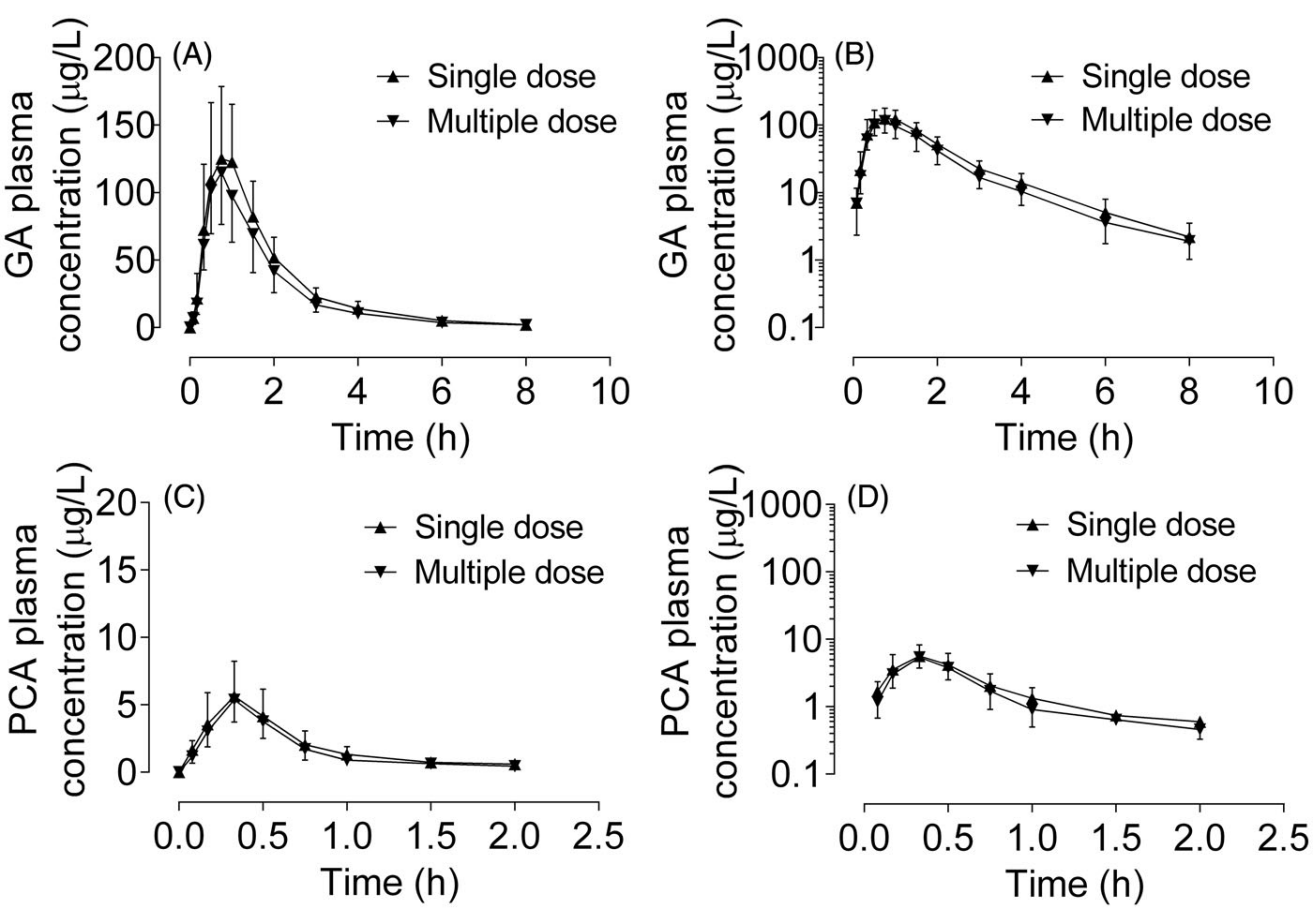
Plasma concentration-time and semilogarithmic scale profiles of gallic acid (A and B) and protocatechuic acid (C and D) after oral administration of single (▲) and multiple (▼) doses of Relinqing granules (RLQ, 8 g) in human subjects (means ± SD, *n* = 12).

GA and PCA showed a relatively high response in human plasma and urine samples, while quercetin, quercetin and kaempferol displayed little or no response. Consequently, GA and PCA were identified as PK-markers of RLQ in this study. Figure 4 presents the plasma concentration-time profiles of GA and PCA after oral administration of single and multiple RLQ doses to human subjects. The main pharmacokinetic parameters of GA and PCA are summarized in Table 4. After a single dose, GA and PCA were rapidly absorbed with the *C*_max_ of 139 ± 49.4 μg/L at 0.75 (0.5–1.0) h and 6.06 ± 2.28 μg/L at 0.33 (0.17–0.5) h, respectively. GA and PCA were rapidly eliminated from the plasma with the *t*_1/2_ values of 1.37 ± 0.37 and 0.33 ± 0.14 h, respectively. The apparent total clearance rates of GA and PCA were 124 ± 42.0 and 337 ± 135 L/h, respectively. No cumulative effects were observed after taking multiple doses, with median accumulation ratios of 0.89 (0.75–1.20) and 1.04 (0.56–1.51) for GA and PCA, respectively.

**Table 4.** Pharmacokinetic parameters of gallic acid and protocatechuic acid after oral administration of single and multiple doses of Relinqing to human subjects (means ± SD, *n* = 6).

Variable	Gallic acid		Protocatechuic acid	
	Single dose	Multiple dose	Single dose	Multiple dose
*C*_max_ (μg/L)	139 ± 49.4	118 ± 39.5	6.06 ± 2.28	5.45 ± 1.58
*AUC*_0–_*_t_* (h·μg/L)	251 ± 74.4	205 ± 62.8	3.20 ± 1.17	2.81 ± 0.81
*AUC*_0–∞_ (h·μg/L)	256 ± 74.9	209 ± 63.5	3.64 ± 1.35	3.09 ± 0.90
*t*_max_ (h)^a^	0.75 (0.5, 1)	0.75 (0.75, 1)	0.33 (0.17, 0.5)	0.33 (0.17, 0.5)
*k*_e_ (h^−1^)	0.53 ± 0.11	0.54 ± 0.12	2.42 ± 0.87	2.84 ± 0.72
*t*_1/2_ (h)	1.37 ± 0.37	1.34 ± 0.32	0.33 ± 0.14	0.26 ± 0.08
*V*_z_/F (L)	241 ± 82.7	292 ± 113	158 ± 101	139 ± 46.1
*CL*/F (L/h)	124 ± 42.0	152 ± 51.6	337 ± 135	379 ± 119
*F*_u_ (%)	8.55 ± 4.62	10.7 ± 7.87	4.24 ± 3.72	5.41 ± 3.40

^a^Median (range).

*AUC*_0–t_: area under the plasma concentration-time curve from time zero to the last measurable concentration; *AUC*_0–∞_: area under the plasma concentration–time curve from time 0 to infinity; *C*_max_: maximum plasma concentration after administration; *CL*/F: total body clearance; *t*_1/2_: terminal elimination half-life; *t*_max_: time of *C*_max_; *F*_u_: fraction of administered dose excreted in the urine; *V*_z_/F: oral volume of distribution

No statistically significant differences were observed between single and multiple doses of RLQ in human using a two-tailed unpaired *t*- test (*p* > 0.05).

### Renal elimination of GA and PCA in human subjects

**Figure 5.**
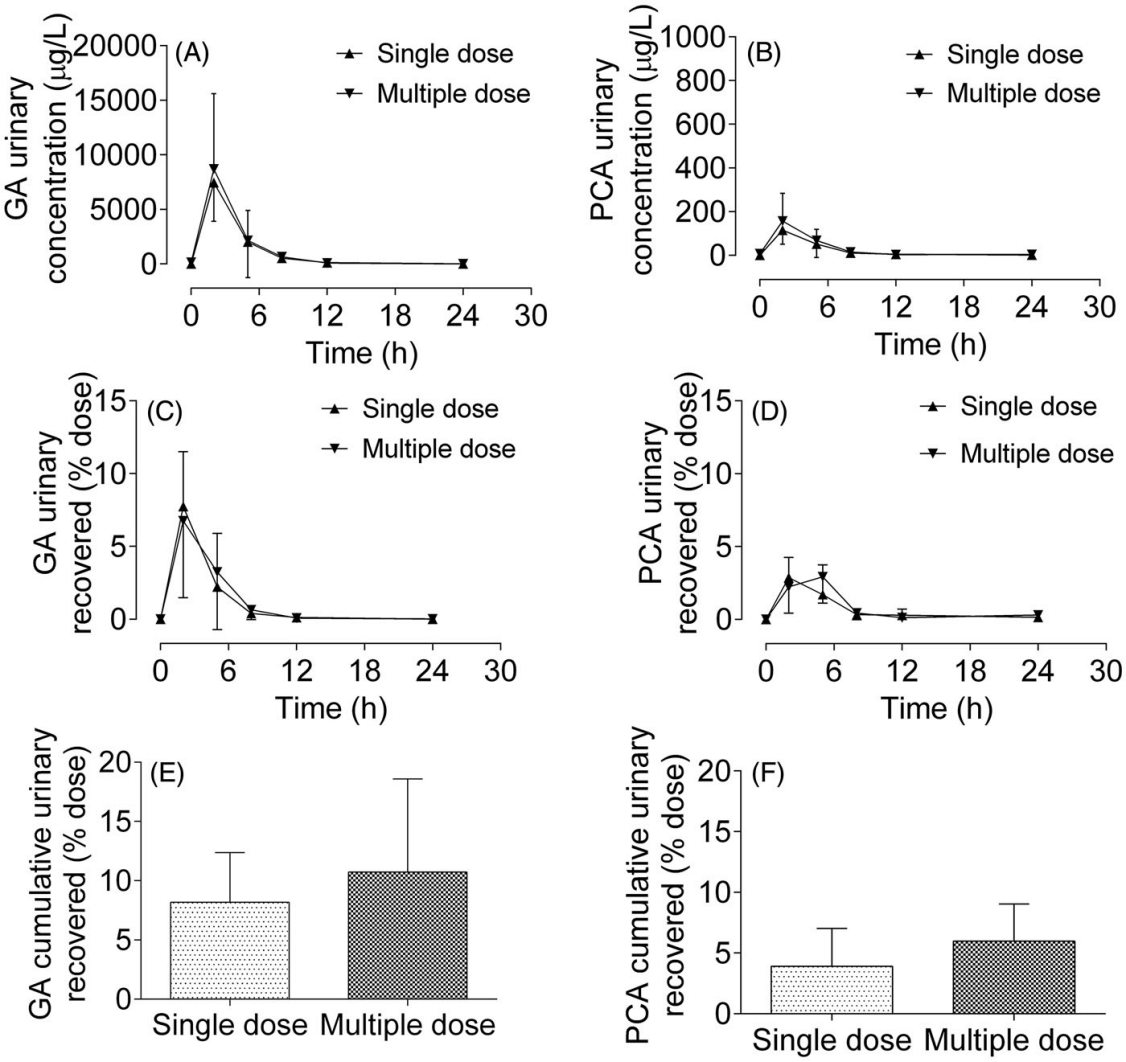
Urinary concentration-time profiles of gallic acid (GA, A) and protocatechuic acid (PCA, B), and urinary recovery-time curves of GA (C) and PCA (D) after oral administration of single (▲) and multiple (▼) doses of Relinqing granules (8 g) to human subjects, as well as the cumulative urinary recoveries of GA (E) and PCA (F) in urine (means ± SD, *n* = 12).

The mean urinary concentration-time profiles of GA and PCA after a single RLQ dose are similar to those observed after multiple doses (Figure 5). The mean urinary concentrations of GA and PCA in the first 2 h after administration were approximately 8500 and 150 μg/L, respectively, indicating that the exposures of GA and PCA in urine were higher than that in plasma. The amounts of GA and PCA recovered in urine within 24 h were 2.48 ± 1.34 and 0.046 ± 0.040 mg, respectively, which suggested that GA and PCA in urine accounted for approximately 8.6% and 4.2% of the total dose, respectively. Meanwhile, the mean fractions of GA and PCA recovered in the urine after multiple doses were 10.73% and 5.41%, respectively. No significant difference was observed in the cumulative excretion of GA and PCA in urine after multiple doses (*p* > 0.05) compared to the single dose.

### Gender-related difference on PK parameters of GA and PCA

**Figure 6.**
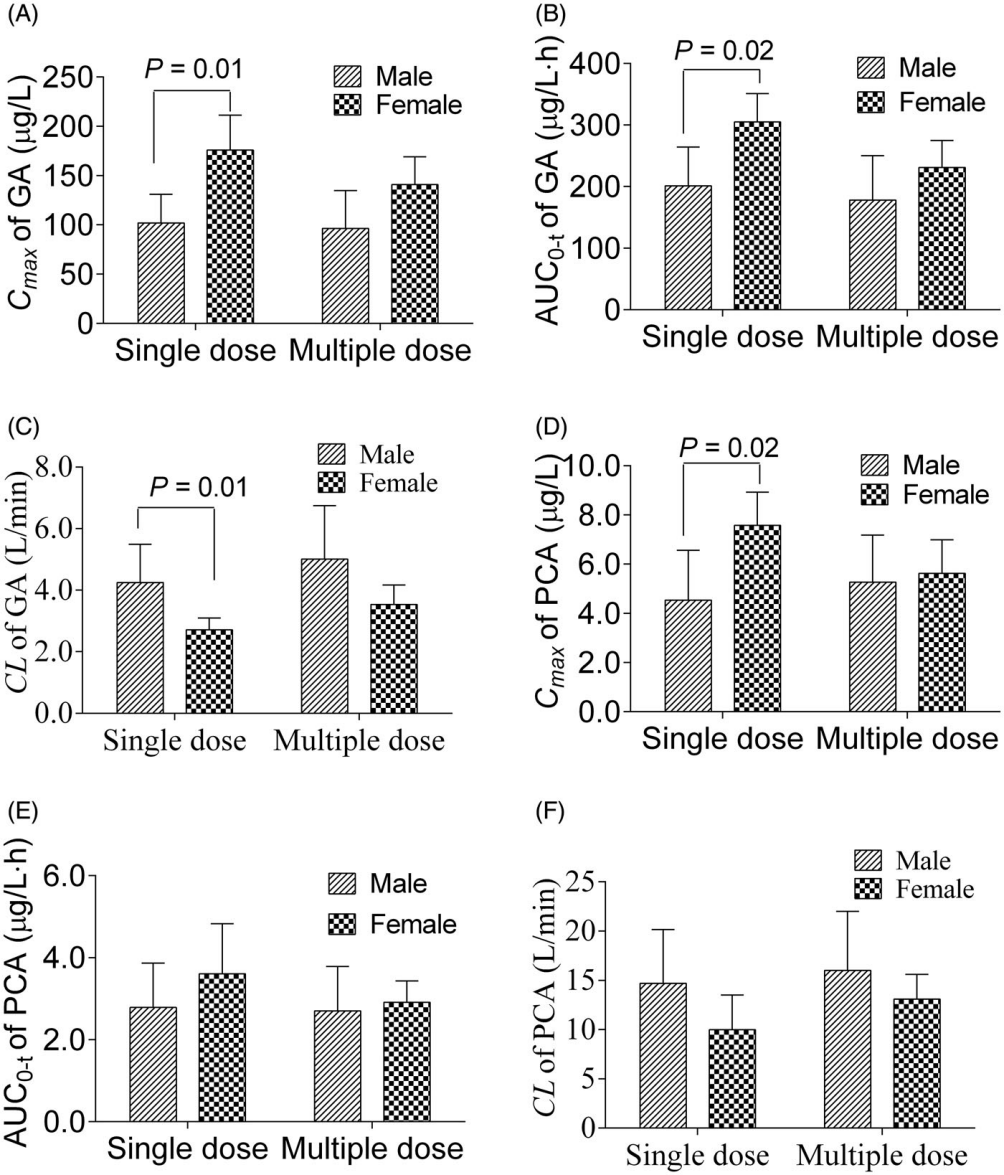
Gender difference on the main pharmacokinetic parameters *C*_max_ (A) and *AUC*_0–_*_t_* (B) of gallic acid, and *C*_max_ (C) and *AUC*_0–_*_t_* (D) of protocatechuic acid after single and multiple doses of Relinqing granules (8 g) in human subjects (means ± SD, *n* = 6).

Gender differences between male and female subjects in the main pharmacokinetic parameters of GA and PCA are compared in Figure 6. After a single RLQ dose, significant gender differences were observed in GA’s plasma pharmacokinetic parameters, including *C*_max_, *AUC*_0–_*_t_* and *CL*/F. Generally, systematic drug exposures are dependent on the volume of distribution and clearance. Compared with men, statistical increases were observed in women in the plasma GA exposures, such as *C*_max_ (102 ± 29.0 and 176 ± 35.3 μg/L in men and women, respectively) and *AUC*_0–_*_t_* (201 ± 63.3 and 301 ± 46.3 h × μg/L in men and women, respectively). The same conclusion was drawn in the plasma PCA exposures with the *C*_max_ of 4.54 ± 2.02 and 7.58 ± 1.35 μg/L in men and women, respectively. Besides, the primary parameters *V*_z_/F and *CL*/F of GA also showed significantly lower levels in females than in males (*p* < 0.05). Since GA and PCA possess a particular affinity with OAT1, OAT3 and OAT4, the pharmacokinetic differences between women and men may be attributed to gender differences in human key transporter activities. We, therefore, conducted transporter-mediated inhibition experiments in the following studies.

### Inhibition of OAT1, OAT3, OCT2 and OATP1B1 mediated uptake by GA and PCA

**Figure 7.**
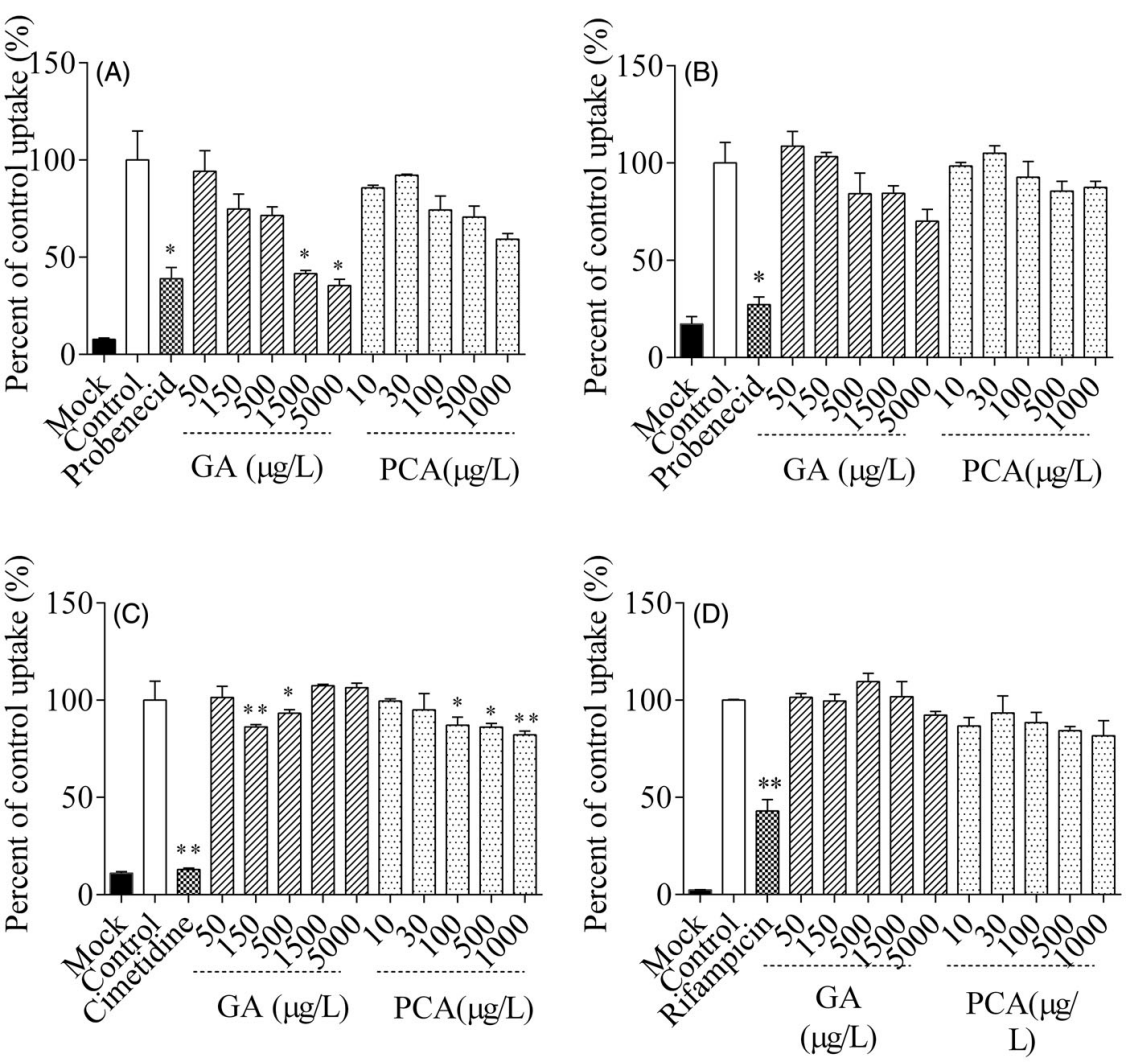
Inhibitory effects of gallic acid (GA) and protocatechuic acid (PCA) on the uptake of ^14^C-amino hippuric acid (^14^C-PAH, A), ^3^H-estrone sulphate-ammonium salt (^3^H-ES, B),^14^C-tetraethylammonium bromide (^14^C-TEA, C) and ^3^H-ES (D) into MDCK-hOAT1, S2-hOAT3, S2-hOCT2 and HEK293-hOATP1B1 cells, respectively.

The net uptake of OAT1-mediated ^14^C-PAH, OAT3-mediated ^3^H-ES, OCT2-mediated ^14^C-TEA and OATP1B1-mediated ^3^H-ES were defined as the control (Figure 7). After co-incubation with GA, the intracellular accumulation of ^14^C-PAH and ^3^H-ES were decreased in the MDCK-OAT1 and S2-OAT3 cells, respectively. In MDCK-OAT1 cells, GA reduced the cellular uptake of ^14^C-PAH to 41.61% and 35.45% of the control at the concentrations of 1500 μg/L (8.82 µM) and 5000 μg/L (29.41 µM), respectively (Figure 6). GA’s inhibiting ability was comparable to probenecid (100 µM, 38.91%), a positive inhibitor of OAT1. The estimated half-maximal inhibitory concentration (IC_50_) of GA was 634.1 μg/L (3.73 µM), suggesting that GA is a potent inhibitor of OAT1. Meanwhile, GA weakly inhibited the OAT3-mediated uptake of ^3^H-ES (an index substrate of OAT3, IC_50_ = 29.41 µM). Compared with the control, GA displayed a significant inhibitory effect on the OCT2-mediated uptake of ^14^C-TEA at concentrations of 150 and 500 μg/L (*p* < 0.05). Similarly, a statistical decline (*p* < 0.05) was observed on the OCT2-mediated uptake of ^14^C-TEA after incubation with PCA at ranges from 100 to 1000 μg/L (0.65–6.5 µM). However, no statistical differences were observed in GA and PCA’s inhibitory effects on the OATP1B1-mediated uptake of 3H-ES. PCA showed no significant inhibitory effect on OAT1 and OAT3 at dose ranges from 10 to 1000 μg/L (0.65–6.5 µM).

### Inhibition of MDR1 and BCRP mediated efflux by GA and PCA

**Figure 8.**
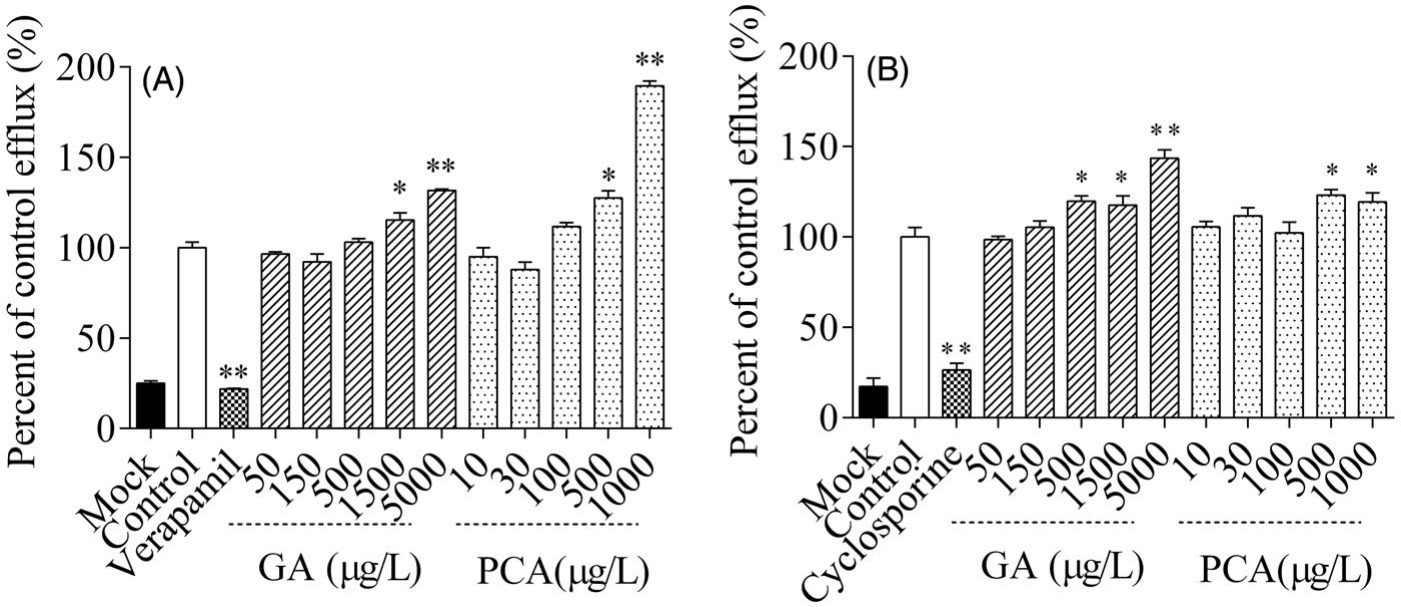
Inhibitory effects of gallic acid (GA) and protocatechuic acid (PCA) on the efflux of rhodamine 123 (A) and lucifer yellow (B) across MDCK-hMDR1 and MDCK-hBCRP cells, respectively.

Inhibition results were demonstrated when MDCK-MDR1 and MDCK-BCRP cells were exposed to GA or PCA within a specific concentration range. After co-incubation with the corresponding positive inhibitor, the cell efflux of rhodamine 123 and lucifer yellow reduced to 21.9% and 26.4%, respectively (Figure 8). However, no significant inhibitory effect was observed on the efflux of MDR1-mediated rhodamine 123 or BCRP-mediated lucifer yellow after co-incubation with GA (50–5000 μg/L) or PCA (10–1000 μg/L), respectively. Compared with the control, a significant increase was presented on the efflux of rhodamine 123 and lucifer yellow after the co-incubation with GA and PCA, respectively, at relatively high concentrations. Especially for PCA at 1000 μg/L (6.5 µM), the efflux of rhodamine 123 rose to approximately 189.5%.

## Discussion

RLQ, a best-selling Chinese patent medicine, is widely used to treat various urological disorders in China. Previous studies have demonstrated that the fractions of phenolic acids and flavonoids are the sugar-free RLQ’s main components (Liao et al. 2013; Zhang et al. 2013). GA has been officially regarded as a quality control ingredient of RLQ to ensure its quality in Chinese Pharmacopoeia (Chinese Pharmacopoeia Commission 2020). In this study, the quantitative analysis of TZQ was performed on five ingredients: GA, PCA, quercitrin, quercetin and kaempferol. Tang et al. (2013) reported that the contents of GA, PCA and quercitrin in eight different batches of RLQ were 26.76–35.06, 0.94–1.27 and 2.88–4.12 mg, respectively. The contents of GA and PCA in this batch of RLQ are comparable to those reported by Tang et al. (2013). The therapeutic effect of RLQ on UTIs is mainly attributed to the antibacterial and anti-inflammatory effects of phenolic acids and flavonoids (Liao et al. 2011). Therefore, GA, PCA, quercitrin, quercetin and kaempferol can safely be used as quality control markers for ensuring the quality assessment and process control of RLQ products.

Due to herbal medicines’ complexity, it is essential to identify suitable PK-markers for illustrating their pharmacokinetic properties. It is the first time single and multiple doses pharmacokinetics of RLQ has been investigated in healthy humans. Before this study, Ma et al. (2015, 2016) selected GA and PCA as PK-markers to depict the *in vivo* disposition and tissue distribution following intragastric administration of 20, 60 and 120 mg/kg RLQ in rats. The plasma exposure parameters (*C*_max_, *AUC*_0–_*_t_* and *AUC*_0–-∞_) of GA and PCA increased proportionally with the increase of RLQ doses from 20 to 120 mg/kg (Ma et al. 2015). The urinary excretion of GA and PCA followed a concentrated elimination over a 4-h period (Ma et al. 2016). However, the amount of unchanged GA and PCA that survived the metabolism was approximately 14.6% and 15.7% of the total intake, respectively (Ma et al. 2016). Whether in humans (Table 4) or animals (Huang et al. 2017), both GA and PCA undergo a rapid absorption process, while the urinary excretions of GA and PCA in humans are lower than that in rats. The reduction may be attributed to species-specific differences in the excretion mechanisms, including the different types and expressions of renal transporters, such as OATs, OCTs and MDR1. GA and PCA exhibited a relatively targeted distribution in kidney tissue (Ma et al. 2016), which helps explain this extract’s practical use as a treatment for kidney diseases. Consequently, GA and PCA have been identified as the preferred PK-markers of RLQ in human plasma and urine.

Extensive reviews on the pharmacokinetic differences between sexes have already been published (Ueno and Hiromi 2012; Mai et al. 2018; Hartigan and Dmochowski 2020). Evidence for the sex-related difference in drug PK/PD is markedly increasing. In many cases, female sex is a risk factor for adverse effects or attenuated clinical responses because of lower clearance, smaller distribution volumes, higher activity of some metabolic enzymes (especially CYP3A4) or presence of sex hormones (Ueno and Hiromi 2012). It is the first study to report gender differences in the pharmacokinetics of GA and PCA. Systematic drug exposures are dependent on the volume of distribution and clearance. GA presented lower clearances, smaller distribution volumes and higher plasma exposures in women than men. Usually, women have a higher percent body fat and a lower rate of glomerular filtration, contributing to the lowered *V*_z_/F of GA. Sex-specific differences in activities of transporters, CYP450, UDGT enzymes and renal excretion will also result in differences in the *V*_z_/F of GA. There is evidence that women have lower CYP1A2, CYP2E1, P-gp and UDGT but higher activities of CYP3A4, CYP2A6 and CYP2B6 (Hartigan and Dmochowski 2020). Mai et al. (2018) reported that P-gp protein and mRNA levels are lower in female rats, contributing to the high bioavailability of the substrate drugs, such as GA and PCA. In brief, the sex-specific differences in GA and PCA pharmacokinetics may be attributed to any process of absorption, distribution, metabolism and excretion. Further study is needed to clarify the mechanism of this phenomenon, which may have considerable pharmacological and clinical relevance.

With the introduction of guidelines on drug interactions, transporter-mediated interactions have assumed increasing value documented in recent years. The secretory transporters OAT1, OAT3 and OCT2 are mainly distributed on the kidney proximal tubules’ basal side. Wang and Sweet (2012) reported that GA and PCA show stronger affinity with OAT1-mediated ^3^H-PAH uptake in CHO-OAT1 cells and OAT3-mediated ES uptake in HEK-OAT3 cells. We have found that GA is a potent inhibitor of OAT1 and a weak inhibitor of hOAT3 and hOCT2, which is roughly consistent with the literature results. The FDA’s Guidance for Industry Drug interaction research recommends that an *in vivo* clinical study is needed where the *C*_max_/IC_50_ ratio of the investigational drug is more than 0.1 (USFDA 2020). The *C*_max_/IC_50_ value of GA was approximately 0.25 in this study, while the value ranged from 0.39 to 1.27 in rat plasma (Ma et al. 2015). The maximum DDI indices for GA indicate that it is necessary to perform *in vivo* DDI studies between GA and sensitive substrates of OAT1. The results have demonstrated that GA-based herbal medicines and foods may significantly alter the pharmacokinetics of OAT1 substrates. A dose-dependent rising trend in the cellular inhibition assay showed statistical increases in the MDR1/BCRP-mediated efflux. The relatively high GA concentrations in human urine (8500 μg/L, Table 4) and rat kidney tissue (1218 ng/g, Ma et al. 2016) will enhance the MDR1/BCRP-mediated transport in the apical side of the proximal tubules of the kidney. In this case, it is necessary to implement an *in vivo* DDI study with an MDR1 or BCRP substrate. Since the co-incubation time is 2 h, it may be challenging to say that the MDR1/BCRP-mediated transport’s statistical increase is due to GA or PCA’s induction effect. Consequently, the mechanism needs further in-depth study.

Although drug therapy using RLQ alone is a feasible and effective treatment in patients with urinary system infection, it is commonly co-administered with antibiotics to enhance UTIs’ treatment (Liao et al. 2011). Ciprofloxacin has been extensively used alone or in combination with other antibacterial drugs in the empiric treatment of infections for which the bacterial pathogen has not been identified (Lu et al. 2016). As reported in the literature, ciprofloxacin is a substrate of transporters OAT1 (Mulgaonkar et al. 2013), OAT3 (Mulgaonkar et al. 2013), MDR1 (Zimmermann et al. 2019), BCRP (Haslam et al. 2011), OATP (Xiao et al. 2014) and OCT2 (Zakelj et al. 2006). The possibility of drug interactions is greatly increased after the combination of RLQ and ciprofloxacin. A previous study has shown that the concomitant administration of RLQ results in an approximately 70% reduction in systemic exposure to ciprofloxacin and an 89% increase in the clearance levels (Lu et al. 2016). The inhibitory effect of GA and PCA on the basolateral uptake transporters OAT1, OAT3 and OCT2 in the proximal tubules of the kidney would seem to increase the systemic circulation of ciprofloxacin (Vanwert et al. 2008), which is not consistent with the 70% reduction in the bioavailability of ciprofloxacin reported by (Lu et al. 2016). Hence the reduction in the plasma ciprofloxacin concentrations would be attributed to GA and PCA’s enhancement effect on the apical efflux transporters MDR1 and BCRP in the enterocyte, hepatocytes and renal tubule cells (Alvarez et al. 2008).

GA and PCA, which have been identified as PK-markers of RLQ, enabled us to study the pharmacokinetic profiles of RLQ and the potential herb-drug interactions between RLQ and concomitant medications. However, some limitations should be acknowledged in this study. Our findings should be interpreted with caution because the recruited subjects were healthy volunteers from the same ethnic background rather than the target patient population from different geographical regions. The effects of GA and PCA on the expressions of the efflux/uptake transporters were examined to explore potential mechanisms of the HDIs, but the two tracer components do not wholly represent the integrated effects of RLQ. Due to herbs’ complexity, some other phytochemicals, such as quercetin, quercetin and kaempferol, may also cause potential HDI between RLQ and concomitant drugs. Wang et al. (2013) reported that quercetin significantly increases MDR1 activity in healthy Chinese subjects. Caco-2 cells pre-treated with quercetin showed an increase in the apical transport of BCRP-mediated benzo(*a*)pyrene-3-sulphate, regulated *via* AhR-dependent signalling pathways (Ebert et al. 2006).

## Conclusions

GA and PCA are identified as PK-markers of RLQ based on the qualitative fingerprint analysis, the multi-component quantitative analysis and the plasma and urine exposure analysis in humans. When herbs rich in GA are used in combination with OAT1 substrate drugs, potential herb-drug interactions may occur.

## References

[CIT0001] Akimitsu M, Shuichi T, Kentarou U, Yoshikatsu K, Hitoshi E, Kazuyuki S, Etsuko M, Akio F. 2010. Drug interaction between celecoxib and methotrexate in organic anion transporter 3-transfected renal cells and in rats *in vivo*. Eur J Pharmacol. 640:168–171. 20478302 10.1016/j.ejphar.2010.04.051

[CIT0002] Alvarez AI, Pérez M, Prieto JG, Molina AJ, Real R, Merino G. 2008. Fluoroquinolone efflux mediated by ABC transporters. J Pharm Sci. 97:3483–3493. 18200507 10.1002/jps.21233

[CIT0003] Benevides Bahiense J, Marques FM, Figueira MM, Vargas TS, Kondratyuk TP, Endringer DC, Scherer R, Fronza M. 2017. Potential anti-inflammatory, antioxidant and antimicrobial activities of *Sambucus australis*. Pharm Biol. 55(1):991–997. 28166708 10.1080/13880209.2017.1285324 PMC6130686

[CIT0004] Chiba S, Ikawa T, Takeshita H, Kanno S, Nagai T, Takada M, Mukai T, Wempe MF. 2013. Human organic cation transporter 2 (hOCT2): inhibitor studies using S2-hOCT2 cells. Toxicology. 310:98–103. 23770354 10.1016/j.tox.2013.06.001

[CIT0005] Chinese Pharmacopoeia Commission. 2020. Pharmacopoeia of the People's Republic of China. Beijing, China: China Chemical Industry Press; p. 1454.

[CIT0006] Ebert B, Seidel A, Lampen A. 2006. Phytochemicals induce breast cancer resistance protein in Caco-2 cells and enhance the transport of benzo[*a*]pyrene-3-sulfate. Toxicol Sci. 96:227–236. 17077187 10.1093/toxsci/kfl147

[CIT0007] Geerlings SE. 2016. Clinical presentations and epidemiology of urinary tract infections. Microbiol Spectr. 4:1–11. 10.1128/microbiolspec.UTI-0002-2012 27780014

[CIT0008] Hartigan SM, Dmochowski RR. 2020. Gender-specific pharmacokinetic and pharmacodynamic considerations for antimuscarinic drugs for overactive bladder treatment. Expert Opin Drug Metab Toxicol. 16:103–110. 31918590 10.1080/17425255.2020.1714591

[CIT0009] Haslam IS, Wright JA, O’Reilly DA, Sherlock DJ, Coleman T, Simmons NL. 2011. Intestinal ciprofloxacin efflux: the role of breast cancer resistance protein (ABCG2). Drug Metab Disp. 39:2321–2328. 10.1124/dmd.111.038323 PMC3226371 21930826

[CIT0010] Huang Y, Sun HY, Qin XL, Li YJ, Liao SG, Gong ZP, Lu Y, Wang YL, Wang AM, Lan YY, et al. 2017. A UPLC-MS/MS method for simultaneous determination of free and total forms of a phenolic acid and two flavonoids in rat plasma and its application to comparative pharmacokinetic studies of *Polygonum capitatum* extract in rats. Molecules. 22(3):353–313. 10.3390/molecules22030353 PMC6155221 28245598

[CIT0011] Huang Y, Zhou Z, Yang W, Gong Z, Li Y, Chen S, Wang Y, Wang A, Lan Y, Liu T, et al. 2019. Comparative pharmacokinetics of gallic acid, protocatechuic acid, and quercitrin in normal and pyelonephritis rats after oral administration of a *Polygonum capitatum* extract. Molecules. 24(21):3873–3813. 10.3390/molecules24213873 PMC6864662 31717895

[CIT0012] Kim C, Ji J, Ho Baek S, Lee JH, Ha IJ, Lim SS, Yoon HJ, Je Nam Y, Ahn KS. 2019. Fermented dried *Citrus unshiu* peel extracts exert anti-inflammatory activities in LPS-induced RAW264.7 macrophages and improve skin moisturizing efficacy in immortalized human HaCaT keratinocytes. Pharm Biol. 57:392–402. 31188689 10.1080/13880209.2019.1621353 PMC6566750

[CIT0013] Klein RD, Hultgren SJ. 2020. Urinary tract infections: microbial pathogenesis, host-pathogen interactions and new treatment strategies. Nat Rev Microbiol. 18(4):211–226. 32071440 10.1038/s41579-020-0324-0 PMC7942789

[CIT0014] Li L, Song F, Tu M, Wang K, Zhao L, Wu XD, Zhou H, Xia ZL, Jiang HD. 2014. *In vitro* interaction of clopidogrel and its hydrolysate with OCT1, OCT2 and OAT1. Inter J Pharm. 465(1–2):5–10. 10.1016/j.ijpharm.2014.02.003 24530383

[CIT0015] Liao SG, Zhang LJ, Sun F, Wang Z, He X, Wang AM, Li YJ, Huang Y, Lan YY, Zhang BL. 2013. Identification and characterisation of phenolics in *Polygonum capitatum* by ultrahigh-performance liquid chromatography with photodiode array detection and tandem mass spectrometry. Phytochem Anal. 24:556–568. 24154994 10.1002/pca.2432

[CIT0016] Liao SG, Zhang LJ, Sun F, Zhang JJ, Chen AY, Lan YY, Li YJ, Wang AM, He X, Xiong Y, et al. 2011. Antibacterial and anti-inflammatory effects of extracts and fractions from *Polygonum capitatum*. J Ethnopharmacol. 134(3):1006–1009. 21296143 10.1016/j.jep.2011.01.050

[CIT0017] Lu Y, Gong ZP, Xie YM, Pan J, Sun J, Li YT, Chen SY, Li YJ, Wang YL, Huang Y. 2016. Herb-drug interaction: effects of Relinqing® granule on the pharmacokinetics of ciprofloxacin, sulfamethoxazole, and trimethoprim in rats. Evid Based Comp Altern Med. 2016:1–6. 10.1155/2016/6194206 PMC5027041 27688790

[CIT0019] Ma F, Gong X, Zhou X, Zhao Y, Li M. 2015. An UHPLC-MS/MS method for simultaneous quantification of gallic acid and protocatechuic acid in rat plasma after oral administration of *Polygonum capitatum* extract and its application to pharmacokinetics. J Ethnopharmacol. 162:377–383. 25557034 10.1016/j.jep.2014.12.044

[CIT0020] Ma FW, Deng QF, Zhou X, Gong XJ, Zhao Y, Chen HG, Zhao C. 2016. The tissue distribution and urinary excretion study of gallic acid and protocatechuic acid after oral administration of *Polygonum capitatum* extract in rats. Molecules. 21:1–14. 10.3390/molecules21040399 PMC6273519 27023501

[CIT0021] Mai Y, Dou L, Murdan S, Basit AW. 2018. An animal's sex influences the effects of the excipient PEG 400 on the intestinal P-gp protein and mRNA levels, which has implications for oral drug absorption. Eur J Pharm Sci. 120:53–60. 29678614 10.1016/j.ejps.2018.04.021

[CIT0022] Millner R, Becknell B. 2019. Urinary tract infections. Pediatr Clin North Am. 66:1–13. 30454735 10.1016/j.pcl.2018.08.002

[CIT0023] Mulgaonkar A, Venitz J, Gründemann D, Sweet DH. 2013. Human organic cation transporters 1 (SLC22A1), 2 (SLC22A2), and 3 (SLC22A3) as disposition pathways for fluoroquinolone antimicrobials. Antimicrob Agents Chemother. 57(6):2705–2711. 23545524 10.1128/AAC.02289-12 PMC3716151

[CIT0024] Qindeel M, Barani M, Rahdar A, Arshad R, Cucchiarini M. 2021. Nanomaterials for the diagnosis and treatment of urinary tract infections. Nanomaterials. 11(2):546–521. 33671511 10.3390/nano11020546 PMC7926703

[CIT0025] Ren Y, Li H, Liu X. 2019. Effects of *Ginkgo* leaf tablets on the pharmacokinetics of atorvastatin in rats. Pharm Biol. 57:403–406. 31188698 10.1080/13880209.2019.1622569 PMC6566491

[CIT0026] Reznicek J, Ceckova M, Ptackova Z, Martinec O, Tupova L, Cerveny L, Staud F. 2017. MDR1 and BCRP transporter-mediated drug-drug interaction between rilpivirine and abacavir; effect on intestinal absorption. Antimicrob Agents Chemother. 61(9):837–817. 10.1128/AAC.00837-17 PMC5571350 28696229

[CIT0027] Tamadonfar KO, Omattage NS, Spaulding CN, Hultgren SJ. 2019. Reaching the end of the line: urinary tract infections. Microbiol Spectr. 7:1–16. 10.1128/microbiolspec.bai-0014-2019 PMC11314827 31172909

[CIT0028] Tandogdu Z, Wagenlehner FM. 2016. Global epidemiology of urinary tract infections. Curr Opin Infect Dis. 29:73–79. 26694621 10.1097/QCO.0000000000000228

[CIT0029] Tang L, Liu Y, Zheng L, Liao SG, Huang Y. 2013. Determination of five components in Relinqing granules by UPLC-MS/MS. Chin J Pharm. 44:1029–1032.

[CIT0030] Ueno K, Hiromi S. 2012. Sex-related differences in pharmacokinetics and pharmacodynamics of anti-hypertensive drugs. Hypertens Res. 35:245–250. 22089536 10.1038/hr.2011.189

[CIT0031] US Food and Drug Administration (FDA). 2020. In vitro drug interaction studies, cytochrome P450 enzyme- and transporter-mediated drug interactions guidance for industry. Rockville (MD): Food and Drug Administration. [accessed 2020 Aug 15]. https://www.fda.gov/media/134582/download.

[CIT0032] US Food and Drug Administration. 2018. Bioanalytical method validation guidance for industry. Rockville (MD): Food and Drug Administration. [accessed 2020 Aug 15]. https://www.fda.gov/media/70858/download.

[CIT0033] Vanwert AL, Srimaroeng C, Sweet DH. 2008. Organic anion transporter 3 (OAT3/SLC22A8) interacts with carboxyfluoroquinolones, and deletion increases systemic exposure to ciprofloxacin. Mol Pharmacol. 74(1):122–131. 18381565 10.1124/mol.107.042853 PMC2822873

[CIT0034] Wang L, Sweet DH. 2012. Potential for food-drug interactions by dietary phenolic acids on human organic anion transporters 1 (SLC22A6), 3 (SLC22A8), and 4 (SLC22A11). Biochem Pharmacol. 84:1088–1095. 22877817 10.1016/j.bcp.2012.07.027

[CIT0035] Wang SY, Duan KM, Li Y, Mei Y, Sheng H, Liu H, Mei X, Ouyang W, Zhou HH, Liu ZQ. 2013. Effect of quercetin on P-glycoprotein transport ability in Chinese healthy subjects. Eur J Clin Nutr. 67:390–394. 23422925 10.1038/ejcn.2013.5

[CIT0036] Xiao Y, Deng J, Liu X, Huang JJ, Sun YX, Dai RK, Hong M. 2014. Different binding sites of bovine organic anion-transporting polypeptide1a2 are involved in the transport of different fluoroquinolones. Drug Metab Disp. 42:1261–1267. 10.1124/dmd.114.057448 24890868

[CIT0037] Yang T, Zheng TH, Zhao Q, Liu W, Li SP, Tao YY, Wang CH, Liu CH. 2020. Effects of Fuzheng Huayu recipe on entecavir pharmacokinetics in normal and dimethylnitrosamine-induced hepatic fibrosis rats. Pharm Biol. 58:1–7. 31847670 10.1080/13880209.2019.1687527 PMC6968529

[CIT0038] Zakelj S, Sturm K, Kristl A. 2006. Ciprofloxacin permeability and its active secretion through rat small intestine *in vitro*. Int J Pharm. 313:175–180. 16529884 10.1016/j.ijpharm.2006.02.004

[CIT0039] Zhang H, Han X, Li Y, Li H, Guo X. 2019. Effects of Danshen tablets on pharmacokinetics of amlodipine in rats. Pharm Biol. 57:306–309. 31060428 10.1080/13880209.2019.1604768 PMC6507817

[CIT0040] Zhang K, Zhang J, Wei S, Jing W, Wang Y, Liu A. 2013. Development and validation of HPLC coupled with triple quadrupole MS for the simultaneous determination of six phenolic acids, six flavonoids, and a lignan in *Polygonum capitatum*. J Sep Science. 36(15):2407–2413. 10.1002/jssc.201300291 23720387

[CIT0041] Zhang S, Huang J, Xie X, He Y, Mo F, Luo Z. 2017. Quercetin from *Polygonum capitatum* protects against gastric inflammation and apoptosis associated with *Helicobacter pylori* infection by affecting the levels of p38MAPK, BCL-2 and BAX. Molecules. 22(5):744–717. 10.3390/molecules22050744 PMC6154337 28481232

[CIT0042] Zheng L, Lu Y, Cao X, Huang Y, Liu Y, Tang L, Liao SG, Wang AM, Li YJ, Lan YY, et al. 2014. Evaluation of the impact of *Polygonum capitatum*, a traditional Chinese herbal medicine, on rat hepatic cytochrome P450 enzymes by using a cocktail of probe drugs. J Ethnopharmacol. 158:276–282. 25446640 10.1016/j.jep.2014.10.031

[CIT0043] Zimmermann ES, de Miranda SC, Neris C, da Silva Torres BG, Schmidt S, Costa TD. 2019. Population pharmacokinetic modeling to establish the role of P-glycoprotein on ciprofloxacin distribution to lung and prostate following intravenous and intratracheal administration to Wistar rats. Eur J Pharm Sci. 127:319–329. 30423435 10.1016/j.ejps.2018.11.007

